# Edible Insect Consumption for Human and Planetary Health: A Systematic Review

**DOI:** 10.3390/ijerph191811653

**Published:** 2022-09-15

**Authors:** Marta Ros-Baró, Patricia Casas-Agustench, Diana Alícia Díaz-Rizzolo, Laura Batlle-Bayer, Ferran Adrià-Acosta, Alícia Aguilar-Martínez, Francesc-Xavier Medina, Montserrat Pujolà, Anna Bach-Faig

**Affiliations:** 1 Faculty of Health Sciences, Open University of Catalonia (UOC), 08018 Barcelona, Spain; 2 School of Health Professions, Faculty of Health, University of Plymouth, Plymouth PL4 8AA, UK; 3 Primary Healthcare Transversal Research Group, Institut d’Investigacions Biomèdiques August Pi i Sunyer (IDIBAPS), 08018 Barcelona, Spain; 4 UNESCO Chair in Life Cycle and Climate Change ESCI-UPF, Universitat Pompeu Fabra, 08003 Barcelona, Spain; 5 elBullifoundation, 08004 Barcelona, Spain; 6 Food Lab Research Group (2017SGR 83), Faculty of Health Sciences, Open University of Catalonia (UOC), 08018 Barcelona, Spain; 7 Unesco Chair on Food, Culture and Development, Open University of Catalonia (UOC), 08018 Barcelona, Spain; 8 Faculty of Agri-Food Engineering and Biotechnology, Universitat Politècnica de Catalunya BarcelonaTech, 08860 Castelldefels, Spain; 9 Food and Nutrition Area, Barcelona Official College of Pharmacists, 08009 Barcelona, Spain

**Keywords:** edible insects, health, sustainability, alternative proteins, planetary health, systematic review

## Abstract

This systematic review aimed to examine the health outcomes and environmental impact of edible insect consumption. Following PRISMA-P guidelines, PubMed, Medline ProQuest, and Cochrane Library databases were searched until February 2021. Twenty-five articles met inclusion criteria: twelve animal and six human studies (randomized, non-randomized, and crossover control trials), and seven studies on sustainability outcomes. In animal studies, a supplement (in powdered form) of 0.5 g/kg of glycosaminoglycans significantly reduced abdominal and epididymal fat weight (5–40% and 5–24%, respectively), blood glucose (10–22%), and total cholesterol levels (9–10%), and a supplement of 5 mg/kg chitin/chitosan reduced body weight (1–4%) and abdominal fat accumulation (4%) *versus* control diets. In other animal studies, doses up to 7–15% of edible insect inclusion level significantly improved the live weight (9–33%), reduced levels of triglycerides (44%), cholesterol (14%), and blood glucose (8%), and increased microbiota diversity (2%) *versus* control diet. In human studies, doses up to 7% of edible insect inclusion level produced a significant improvement in gut health (6%) and reduction in systemic inflammation (2%) *versus* control diets and a significant increase in blood concentrations of essential and branched-chain amino acids and slowing of digestion (40%) *versus* whey treatment. Environmental indicators (land use, water footprint, and greenhouse gas emissions) were 40–60% lower for the feed and food of edible insects than for traditional animal livestock. More research is warranted on the edible insect dose responsible for health effects and on environmental indicators of edible insects for human nutrition. This research demonstrates how edible insects can be an alternative protein source not only to improve human and animal nutrition but also to exert positive effects on planetary health.

## 1. Introduction

There is an urgent need to redesign food systems to improve human and planetary health [1]. It is likely that food systems are already operating beyond some planetary boundaries [2,3,4]. Therefore, more environmentally friendly but also affordable, healthy, and safe approaches need to be adopted to feed the expanding human population [5], which is projected to reach 9.7 billion by 2050 [6]. One of the major challenges is to re-align future protein supply and demand, especially animal protein [7], which is expected to rise by 70–80% between 2012 and 2050 [8]. Underutilized plants, insects, and single-cell organisms (e.g., algae, fungi, and bacteria) as well as cultured meat are being considered as novel protein sources to sustainably meet future global requirements [9,10].

Although insects have been consumed since early in human evolution, a new trend in food science began in 2013, when the Food and Agriculture Organization of the United Nations (FAO) pointed out the need to examine modern food science practices to increase the trade, consumption, and acceptance of insects [11]. In regulation 2015/2283 [12] of the European Parliament and the Council of the European Union, whole insects and their parts were included in the category of novel foods. Furthermore, in 2015, the European Food Safety Authority (EFSA) provided a scientific opinion on insect consumption and suggested a list of insect species with high potential use as food for animal feed and human food [13,14]. In 2021, the EFSA issued a positive opinion on the safety of dried yellow mealworm—*Tenebrio mellitus larvae* (*TM larvae*) [13], *Locusta migratoria (LM)* [15], and *Acheta domesticus (AD)* [16]—as a novel food according to European Union regulation 2015/2283 [17]. From a nutritional point of view, edible insects are being proposed as an alternative source of protein for humans and animals [18] due to their high levels of essential amino acids (EAA), unsaturated fatty acids, micronutrients (e.g., vitamin B12, iron (Fe), zinc, and calcium), and fiber [19]. Furthermore, edible insects have various bioactive compounds in their composition with potential health effects [20]. 

Previous systematic reviews on edible insects have focused on studying their nutritional composition [19,21,22], the presence of viruses [23], their effect on human and animal health [24,25,26], and allergic risks [27]. However, the global impact of edible insects on health and the environment remains to be elucidated. Previous reviews on health outcomes centered on either humans or animals and did not adopt a comprehensive approach. In the present review, data were retrieved from human studies on all relevant health outcomes (changes in growth, blood parameters, gut microbiome, changes in muscle mass composition, etc.), on the grams of edible insect, on the insect or part of insect used, and on the insect inclusion level. The aim of this systematic review was to provide an overview of human trials and animal studies to evaluate the effect of edible insect supplementation on health outcomes as well as studies on the environmental impact of edible insects as an alternative and more sustainable source of protein for humans and animals.

## 2. Material and Methods

### 2.1. Search Strategy 

We conducted a systematic review in accordance with the Preferred Reporting Items for Systematic Reviews and Meta-Analyses guidelines [28] and registered it in PROSPERO (https://www.crd.york.ac.uk/prospero/ (accessed on 3 June 2021)) for humans (CRD42021243673) and animals (CRD42021243772). Following the PRISMA-P checklist, studies were identified by the electronic search of three databases (PubMed, ProQuest Medline, and Cochrane Library) for studies published between October 2010 and 28 February 2021. Combinations of the following search terms were used: “GHG”, “greenhouse gas emission”, “environmental impact”, “environmental”, “sustainability”, “sustainable”, “water use”, “phosphor emission”, “land use”, “nitrogen emission”, “eco-friendly”, “climate-friendly”, “life cycle assessment”, “sustainable”, “alternative animal-source”, “entomophaga”, “insect”, “insecta”, “insects”, “edible”, “consumption”, “nutrition”, “supplementation”, “protein”, “health”, and “complementary”. Boolean connectors (AND, OR) were used to search for associations between these terms.

### 2.2. Eligibility Criteria 

Studies were eligible for inclusion if they were human investigations (experimental studies, randomized and controlled trials, and observational studies such as cohort, cross-sectional, and case-control studies) or investigated animal consumption of edible insects (placebo or reference treatment) reporting data on health and sustainability. The review also included ecological studies that evaluated greenhouse gas emission (GHG), water footprint (WFP), land use (LU), and/or energy use (EU) as environmental indicators and those that assessed the feed conversion ratio. We excluded edible insect studies on nutrition composition, acceptance, food technology, gastronomy, allergy, and toxicology. Systematic reviews, meta-analyses, and cell culture, in vitro, and ex vivo studies were all excluded. 

### 2.3. Study Selection and Data Extraction

Search results were downloaded to EndNote (Clarivate Analytics, Philadelphia, PA) and duplicates were removed. Titles and abstracts were screened in duplicate by two of three authors (M.R.-B., P.C.-A., and A.B.-F.) for eligibility. The third author resolved disagreements. Full texts were obtained for any article that appeared to meet eligibility criteria.

Information was extracted from the animal studies on: author(s), year and country of publication, type of animal, sample size, sex, and age, length of intervention(s) (days), edible insect use, number of intervention groups with sample sizes, insect inclusion rate of complementary food product (CFP) (g/100 g expressed in %), variables/outcome, and evaluated health parameters.

Information was extracted from the human studies on: author(s), year and country of publication, sample size, sex, and age, length of intervention(s) (days), edible insect use, intervention groups with sample sizes, daily food portion of intervention with insects (g), insect inclusion rate of CFP (g/100 g expressed in %), insect inclusion level of CFP (expressed in g) per each age group, protein inclusion level of CFP per day (expressed in g), variables/outcomes, and evaluated health parameters.

Information was extracted from the sustainability articles on: GHG (Kg CO_2_), which falls under the indicator global warming potential equivalent (GWP), EU (MJ) as a measure of fossil fuel depletion, LU (m^2^), for the amount of arable land used in the production chain, and finally the WFP (m^3^). The environmental impact was subsequently coupled with a functional unit (FU), a quantitative measure indicating the function of a product. For insects, FUs were expressed in kilograms of protein [29]. The environmental impacts of different steps within the system border were added together to express the total impact on certain environmental indicators. Finally, the total impact was divided by the number of FUs to yield the environmental impact per FU, which was used to compare environmental indicators between similar food products. 

### 2.4. Quality Assessment

Quality and risk of bias were assessed using the Syrcle’s risk of bias tool [30] for preclinical animal studies and the Cochrane risk of bias tool [31] for human studies. Both tools covered the following bias domains: selection bias (random sequence generation and allocation concealment), performance bias (blinding of participants and personnel), detection bias (blinding the outcome assessment), attrition bias (incomplete outcome data), reporting bias (selective reporting), and others. According to the score obtained, studies were classified as having a low, high, or unclear risk of bias (Table 1).

Limitations of this review include gaps in the data available on the nutritional composition and quantity of edible insects administered in human studies and on the form of their administration, and some of this information could only be obtained after contacting the authors. Few studies specify the metamorphic phase of the edible insect, hampering comparisons of the % protein and composition of the complementary food product. It was also sometimes difficult to determine the stage at which values were assigned (e.g., farm gate or mill gate) and to gather information on the diet fed to the edible insects. 

## 3. Results

### 3.1. Literature Search Results

**Figure 1:**
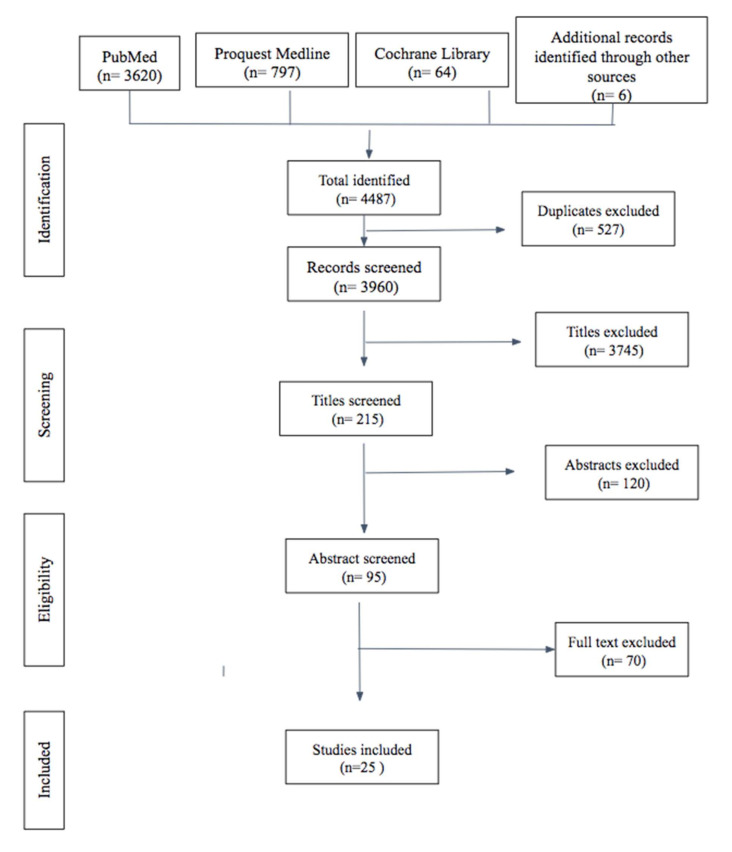
Flow chart of the selection of reviewed articles.

The database search retrieved 4487 articles. After removing duplicates, the titles and abstracts of 3960 articles were assessed independently and in duplicate by two investigators. Eligibility criteria were finally met by 25 studies, which were included in the present systematic review (Figure 1). Table 2, Table 3 and Table 4 summarize the findings of these studies, categorized as animal [32,33,34,35,36,37,38,39,40,41,42,49], human [43,44,45,46,47,48], or sustainability [7,50,51,52,53,54,55] studies. 

### 3.2. Health Outcomes in Animal Studies 

Study outcomes were related to **appetite control** [32], **growth performance** [34,35,38,39,49], **metabolic traits** [32,33,34,35,36,38,39,40,41,42], **crude protein digestibility** [36,38], and/or **intestinal morphology** [35,37,38]. The following seven edible insects were investigated as a supplement in animal studies: *TM* [33,37,38,40], *Hermetia illucens* (*HI*) [35,36,38], *Gryllodes sigillatus* (*GS*) [34,49], *Gryllodes bimaculatus (GB)* [41,42], *Allomyrina dichotoma* larvae (*ALLD*) [32], *Rhynchophorus phoenicis fabricius (RF)* [39], and *AD* [39]. Nine studies used whole insects and three used insect components such as chitin [42], chitosan [49], or glycosaminoglycan [41,42] (Table 2).

#### 3.2.1. Appetite Control

Kim et al. [32] studied the effect of 10 mg/mL of ethanol extract of *ALLD* larvae on the anorexigenic and endoplasmic reticulum (ER) and the stress-reducing effects of *ALLD* on the hypothalamus of previously diet-induced obese mice. Intraventricular cannulation was used to infuse 1 μL of 20% dimethyl sulfoxide (DMSO) and 1 μL of *ALLD* extract (10 mg/mL). Results showed that administration of *ALLD* extract significantly reduced food intake and body weight via appetite-related neuropeptide regulation for 24 h compared to DMSO, which was evident at 2 h after infusion and consistent after 24 h.

#### 3.2.2. Growth Performance 

Dabbou et al. [35] evaluated the effects of increasing levels of partially defatted *HI* larva meals on growth performance in 256 male broiler chickens over 35 days. The diets included increasing levels of *HI* larva meal (0, 5, 10, and 15%; *HI*0, *HI*5, *HI*10, and *HI*15, respectively), with *HI0* as control diet. Increasing levels of dietary *HI* meal administration (5% to 15%, expressed as insect inclusion level of CFP) significantly improved the growth performance (live weight and daily feed intake) of birds by up to 10% in the starter period. The same outcome was reported by Gasco et al. [38] and later studies with replacement meals of *HI* and *TM.* Gasco et al. replaced soybean oil with *HI* and *TM* to meet the growth requirements of 200 36-day-old crossed rabbits for 41 days. Five interventions were tested, with a control diet of 1.5% soybean oil, the partial (50%) or total (100%) substitution of soy-bean oil by *HI (HI*50 and *HI*100, respectively) or by *TM (TM*50 and *TM*100, respectively). *HI* and *T* fats are suitable sources of dietary lipid in rabbit diets to replace soybean oil and have no detrimental effect on growth performance. A study by Agbemafle et al. [39] analyzed the effect of edible insect powder (*AD* and *RF*) on the nutritional status of malnourished rats using the hemoglobin/protein repletion method in 66 21-day-old male rats for 35 days. Malnutrition was induced by feeding the rats with a 5% protein and ~2 ppm Fe diet for 21 days. Results showed similar increases in weight, bone mineral content, and lean and fat mass in the *AD + Solanum torvum, AD,* and *RF* groups in comparison to the protein-Fe sufficient group. Bergmans et al. [34] examined the impact of protein-malnutrition and subsequent recovery on body weight and selected inflammatory biomarkers in a study of 65 3-week-old mice for 66 days. Protein malnutrition was induced by administration of an isocaloric hypoprotein diet (5% protein calories) in young male mice for two weeks, followed by a six-week recovery period using a cricket- (*GS*), peanut-, or milk-based diet. The cricket-based diet performed as well as peanut- and milk-based diets in body weight recovery (34%, 39%, and 32%, respectively). In relation to growth performance, Lokman et al. [49] used parts of the insects, evaluating and comparing the effect of dietary chitin and chitosan from cricket and shrimp on growth performance, carcass quality, and organ characteristics in 150 broiler chickens. The authors observed that cricket chitin at 0.5 g/kg significantly improved growth performance, carcass quality, and organ characteristics of broilers in comparison to the chitosan and control diets, which produced a comparatively greater accumulation of fat.

#### 3.2.3. Metabolic Traits 

In relation to the **body weight gain achieved with** edible insects, Seo et al. [33] investigated the lipid accumulation and anti-obesity effects of whole powder of *TM* larvae with a diet that simultaneously induced obesity in 35 male mice. In this intervention, five treatment conditions were assigned for 6 weeks The body weight gain of the mice was significantly reduced by up to 19% with the oral administration of 100 mg/kg *TM* larvae and by 25% with 3000 mg/kg *TM* larvae in comparison to mice fed with high-fat-diet (HFD) alone. Ahn et al. [42] investigated the effect of glycosaminoglycan from *GB* (*GbG*) on anti-atherosclerotic and antilipidemic effects (including weights of abdominal and epididymal fat) in 50 14-week-old male rats. The rats were acclimated for 1 week with an HFD (60% fat) and then segregated into five treatment groups (control, 5 mg/kg *GbG*, 10 mg/kg *GbG*, 2 mg/kg Pravastatin, and 10 mg/kg chitosan) of 10 rats each. Each group was maintained on the HFD for 1 month. Abdominal fat weight and epididymal fat were significantly decreased in comparison to controls by 16% and 18%, respectively.

In terms of the effects of edible insects on **inflammation**, Ahn et al. [42] also investigated the effect of *GbG* on serum C-reactive protein (CRP) levels. Significant decreases in CRP levels (mg/L) of the *GbG*-treated groups were observed *versus* controls. Furthermore, Bergmans et al. [34] analyzed the gene expression of several inflammatory (TLR4, TNFα, IL-1β, and IFNγ) and anti-inflammatory (IL-4) markers in spleen tissue, observing a similar expression of inflammatory genes in mice on cricket- and milk-based diets to that in mice on a control diet. Both articles by Ahn et al. [41,42] showed that treatment with 5 mg/kg *GbG* reduced glucose levels *versus* controls. Serum aspartate transaminase (AST) and alanine transaminase (ALT) levels were also reduced after *GbG* treatment [41,42]. *TM* larvae also significantly decreased the accumulation of hepatic lipid droplets and levels of plasma ALT and AST in comparison to mice fed with HFD [33]. In another study, the inclusion of insect lipids in rabbit diets did not influence serum AST, ALT, or alkaline phosphatase (ALP) enzyme activities [38]. 

The **antioxidant** effect of edible insect intake has been addressed by various authors [35,41]. Dabbou et al. [35] observed increasing plasma glutathione peroxidase (GPX) activity in the *HI* groups, which showed a linear response (*p* = 0.002) to an increasing percentage of *HI* meal up to 15%. Ahn et al. [41] studied the antioxidative effects of field cricket *GbG* on two types of male diabetic mice at 12 weeks of age: heterozygous (db/+) (DB-Hetero, normal) and homozygous (db/db) (DB-Homo, diabetes) animals. Results showed that the intake of 5 mg/Kg *GbG* significantly increased the anti-oxidative activities of catalase, superoxide dismutase (SOD), and GPX in the *GbG*-treated group *versus* controls (DB-Homo), observing a reduction in hepatocellular biomarkers after 5 mg/kg of *GbG* treatment. Levels of antioxidative enzymes and activities of catalase, GPX, glutathione-s-transferase, and SOD were also increased by the *GbG* treatment. In this way, hepatocellular oxidative stress triggered by free radical damage was attenuated by these antioxidant enzymes. In the db mice experiment, 5 mg/Kg *GbG* increased catalase activity by 114.9%, GPX by 248.1%, GST by 117.6%, and SOD by 125.7%. In terms of blood cell oxidative damage, protein oxidative damage was also reduced by these GAGs (CaG5 by 18.5%; 5 mg/Kg *GbG* by 18.5%; and Metformin10 by 7.0%), based on the blood neutrophil carbonyl content.

In terms of the effects of edible insects on **blood pressure**, Pessina et al. [40] studied the effects of the protein obtained from the larval stage of *TM* on 24 male spontaneously hypertensive rats (SHRs) and 18 male age-matched rats of the normotensive Wistar Kyoto strain (WKY). Results showed that both the standard diet supplemented with *TM*s and Captopril significantly reduced systolic blood pressure, heart rate, and coronary pressure in SHRs compared with the standard diet. Ahn et al. [42] observed no statistically significant differences in blood pressure (systolic blood pressure and heart rate) between the 5 or 10 mg/kg *GbG* treated groups and controls.

Regarding the effect of edible insect intake on the **blood lipid profile**, Bovera et al. [36] studied the effect of replacing 25% or 50% of soybean content with *HI* larvae meal on 162 16-week-old laying hens for 140 days. A reduction in serum cholesterol and triglyceride levels was observed in both insect-meal fed groups. In a mouse study, Bergmans et al. [34], observed that a recovery diet with cricket (*GS*) for 6 weeks reduced serum levels of triglycerides by 47% in comparison to the control diet. Ahn et al. found a reduction in total serum cholesterol after *GbG* treatment *versus* controls [42] in one study and an inhibition of serum LDL-cholesterol levels after *GbG5* treatment in another [41].

In terms of **other relevant blood parameters**, Agbemafle et al. [39] studied the effects of protein-Fe supplementation on hemoglobin in rats. Hemoglobin iron did not significantly differ among protein-Fe sufficient, *AD*, and *RF* groups. Hemoglobin iron was lowest for the *Solanum torvum* and low protein-Fe groups but highest for the control group. Hemoglobin iron was similar among the low protein-Fe, cricket + *Solanum torvum*, and *RF* groups. Out of all supplemented groups, the cricket + *Solanum torvum* evidenced the greatest change in hemoglobin iron, although this did not differ from the *RF* or protein-Fe sufficient groups. Both studies by Ahn et al. [41,42] showed that treatment with 5 mg/kg *GbG* reduced glucose levels *versus* controls. 

#### 3.2.4. Crude Protein Digestibility

Gasco et al. [38] studied crude protein digestibility in rabbits. The addition of *HI* and *T* fats did not influence protein digestibility. In a study of hens, Bovera et al. [36] showed that dry matter, organic matter, and crude protein digestibility coefficients were lower after the *HI*50 diet than after the HI25 diet, probably due to the negative effect of chitin. The dry matter consisted of all nutrients, whereas the organic matter consisted of all nutrients except ash. The crude protein digestibility coefficient is expressed as % of g protein digested per Kg dry matter [36]. 

#### 3.2.5. Intestinal Morphology

Biasato et al. [37] evaluated the effects of *TM* meal for 43 days on the intestinal microbiota, morphology, and mucin composition of 70 female free-range chickens. Chickens received a corn-soybean gluten meal-based control diet or a 75 g/kg *TM diet* in complete substitution of corn gluten meal. Inclusion of the *TM* dietary meal had no effect on intestinal morphometric indices of the free-range chickens (*p* > 0.05) or on mucin staining intensity of intestinal villi but had a significant effect on gut segment and villus fragment histochemistry (*p* < 0.001 and *p* < 0.01, respectively). Gasco et al. [38] observed that villi height and crypt depth ratio were similarly affected after the dietary inclusion of *HI* and *T* fats *versus* controls. In another study, Dabbou et al. [35] found a lower villus height and greater crypt depth in groups receiving meals with 15% *versus* 0–10% *HI* inclusion rates. 

### 3.3. Health Outcomes in Human Studies

The outcomes of studies were classified as **growth performance** [43,44,45], **metabolic traits** [43,44,46], **gut microbiota composition** [46], *changes in muscle mass composition and strength* [47], and **crude protein digestibility** [48]. Five different edible insects were investigated in human studies, including *AD* [47,48], *Haplopelma species (HP)* [43], *Caterpillar* (*CT*) [44], *Rhynchophorus ferrugineus* (*RF*) [45], and *Gryllodes sigillatus* (*GS*) [46]. A parallel design was used by four studies, a randomized design by three [43,44,47] and a non-randomized trial by one [45], while two were cross-over randomized trials [46,48]. Skau et al., 2015, Bauserman et al., 2015, and Nirmala et al., 2017 use the whole edible insect whereas Stull et al., 2018 and Vangsoe et al., A 2018 used powdered form and Vangsoe et al., B 2018 used an isolated protein form. 

#### 3.3.1. Growth Performance

Skau et al. [43], used a single-blinded parallel design to study the effect of two rice-based complementary food products (one containing edible spiders) for 9 months on body composition fat-free mass (FFM) and linear growth in 419 six-month-old Cambodian infants. No significant differences were found in FFM or anthropometric changes (weight, height, knee–heel length) between locally produced products (WF and WF-L) and the CSBs. In a cluster randomized controlled trial, Bauserman et al. [44] assessed the efficacy of a cereal made from caterpillars, a micronutrient-rich, locally available alternative animal-source food, to reduce stunting and anemia in 222 infants. Using a non-randomized controlled trial, Nirmala et al. [45] investigated the effect of sago worm *RF* consumption as a component of complementary feeding *versus* a control diet without sago worms for 45 days on the weight and height of 23 infants aged 1–5 years old. No between-group differences in weight or height were observed in the last two studies [44,45].

#### 3.3.2. Metabolic Traits

The effects on metabolic traits were studied by Skau et al. [43], who observed no significant differences in iron status (plasma ferritin, soluble transferrin receptor (sTfR), or hemoglobin concentration) between locally produced products (WF and WF-L) and corn-soy blends. In another study by Bauserman et al. [44], higher Hb concentrations were found in infants in the *caterpillar* cereal group than in the control group (10.7 vs. 10.1 g/dL, *p* = 0.03), and fewer infants were anemic (26% vs. 50%). In a double-blinded randomized crossover study, Stull et al. [46] investigated the effects of 25 g/day of whole cricket powder in 20 healthy adults aged 18–65 years. Participants were randomized into two study arms and consumed either cricket-containing or controlled breakfast foods for 14 days, followed by a washout period and assignment to the opposite treatment. Blood and stool samples were collected at baseline and after each treatment period to assess liver function and microbiota changes. Results evidenced an association between cricket consumption and reduced plasma tumor necrosis factor-alpha (TNF-α).

#### 3.3.3. Gut Microbiota Composition

Results of the aforementioned study by Stull et al. [46] showed that consuming 25 g/day of whole cricket powder supported the growth of probiotic bacterium, *Bifidobacterium animalis*, which underwent a 5.7-fold increase.

#### 3.3.4. Changes in Muscle Mass Composition and Strength

The effect of insect protein as a dietary supplement on muscle mass and strength during prolonged resistance training was assessed in healthy young men using a randomized, controlled, single-blinded trial [47]. Vangsoe et al. [47] studied the effect of insect protein as a dietary supplement to increase muscle hypertrophy and strength gain during prolonged resistance training in 18 healthy young men. Supplementation with insect protein isolates enhanced muscle mass and strength gains in young men during progressive resistance training, without observing significant differences with those consuming an isocaloric carbohydrate supplement.

#### 3.3.5. Crude Protein Digestibility

In a second study, Vangsoe et al. [48] investigated whether their previous observation of no effect of insect protein on muscle mass gain during training [47] could be explained by the bioavailability, digestibility, and amino acid (AA) profile of the insect protein. Participants received three different protein supplementations (25 g of crude protein from whey, soy, insect) or placebo with water on four separate days. Blood samples were collected at 0, 20, 40, 60, 90, and 120 min during each intervention day. Ingestion of whey, soy, or insect protein isolate was found to produce a significant increase in EAA, branched-chain amino acids (BCAAs), and leucine in comparison to placebo. However, ingestion of whey protein isolate led to significantly higher concentrations of AAs compared with soy or insect protein. Insect protein intake showed a tendency towards higher AA concentrations beyond the 120 min period, suggesting that differences in blood AA concentrations between soy and insect protein may be attributable to a slower digestion of the latter. Furthermore, serum insulin concentrations were significantly increased after ingestion of whey and soy protein but were not changed to the same degree after ingestion of insect protein [48].

### 3.4. Environmental Impacts of Edible Insects

Seven articles assessed the environmental impact of producing insect-based food products for human consumption [7,55] and animal feed; distinguishing between so-called waste-feed insects [50,51,52] and non-waste-feed insects [53,54]. Regarding the type of insects, three articles investigated *TM* larvae [7,53,55], two *HI* larvae [51,52], one *AD* [54], and one *Musca domestica (MD)* [50].

All seven studies applied the life cycle assessment (LCA) methodology to estimate the potential impacts of producing larvae meals. The most common environmental impacts reported were GHG, LU, and EU, with only one article assessing WFP [55]. Table 4 exhibits the environmental impacts per kg of edible protein from the selected studies. In articles that did not report the environmental impacts per kg of edible protein, these were estimated according to the method of Oonincx et al. [7]. First, kg of fresh product was multiplied by the average dry matter (DM) content of the species and the average content of crude protein in the DM. This value was then multiplied by the edible portion, which was considered to be 100% of edible insects. These environmental impacts were also related to the insect production, meaning that the system boundaries of these studies are from cradle-to-insect farm gate [7,51,52,54,55]. Only Thévenot et al. [53] and Van Zanten et al. [50] reported values up to the mill gate.

The highest EUs between cradle and farm gate were related to heating and air-conditioning systems [51]. When the system boundary was extended to the mill gate, the EU increased by around 5–7% [50,53]. LU was related to the land needed for animal farming as well as for crop cultivation to feed the insects. This parameter was minimized in vertically grown insects. LU and GHG emissions are also lower for waste-feed insects than for non-waste feed insects. With regard to water use, cleaning measures were responsible for the largest fraction, and it was also related to process-specific inputs such as substrate rewetting or water provision for drinking, EU (through the water requirements of power plants), and infrastructure construction [50].

Van Zanten et al. [50] explored the environmental impact of using larvae of the common housefly grown on poultry manure and food waste as livestock feed. Likewise, Salomone et al. [51] applied the LCA to a system of mass-rearing of *HI* grown on food waste [51], while Muys et al. [52] used brewery wastes. Thévenot et al. [53] and Halloran et al. [54] used a mixed diet in the group of non-waste-feed insects. In both studies, all LCA indicators were increased in comparison to non-waste feed insects. In particular, Halloran et al. [54] pointed towards a future, more efficient, cricket farming scenario in which environmental impacts could be reduced (e.g., by around 34% for GHG emissions). Oonincx et al. [7] conducted an LCA study on mealworm production for human food in which GHG production, EU, and land use were quantified and compared to conventional sources of animal protein. All parameters were increased in comparison to insect farming for animal feed and especially waste-feed insect farming [50,51,52]. Miglietta et al. [55] evaluated the WFP of the production of edible insects, focusing on the water consumption associated with protein content to allow comparison with other animal protein sources. The results showed a decrease of around > 50% in this resource in comparison to beef and pork and of around < 15% in comparison to chicken.

## 4. Discussion

Food systems are currently facing unprecedented challenges. Rapid depletion of natural resources, climate change, and biodiversity loss further threaten future food systems [56]. Global reports emphasize the need for fundamental transformations of food systems for planetary health [8,57]. 

At first, insects were mainly appreciated for their nutritional composition. Their newly discovered bioactive compounds may promote animal and human health and position insects beyond the ‘simple’ protein concept [58].

With regards to the health dimension, weight-control animal studies found that the inclusion of 1 g/mL ethanol extract to *ADLL* larvae [59,60] reduced food intake and body weight compared with vehicle control (evident at 2 h after infusion and consistent after 24 h) and could be a novel potential treatment option for anorexigenic function in high-fat-induced obese mice via reduction of ER stress. 

Many edible insect species convert organic substrates into protein- and energy-rich products, contributing to circular economy principles [61]. Malnutrition is a major consequence of fragility and is associated with increased mortality, poor cognitive and motor development, impaired physical performance, reduced income in adulthood, and lower birth weight of offspring [44]. Micronutrient deficiencies are a prevalent problem in low-income countries, responsible for 3.1 million deaths annually in children aged <5 years [62], and could be solved in an accessible manner by insect supplementation containing proteins, essential fatty acids, and micronutrients such as riboflavin, pantothenic acid, biotin, and in some cases, folic acid, copper, iron, magnesium, manganese, phosphorus composition, selenium, and zinc, improving growth and nutritional status during childhood [63]. There are two main reasons for using insects in low-income countries: one is to fight malnutrition, and the other is to improve micronutrient iron deficiency and consequently low values of serum hemoglobin and ferritin. As an example, a study by Agbemafle et al. [39] in rats showed an increase in hemoglobin and ferritin concentrations after an *AD* and *RP* diet compared with a normal diet of casein and ferrous sulfate. Superior results were observed in the groups supplemented with the edible insect *AD* with 23.3% protein CFP *versus* controls receiving only a low-protein and iron supplement and the *Solanum torvum* group, which included 8.8% protein. In a study by Skau et al. [43], supplementation for 18 months with cereals containing insect flour (23%) increased plasma hemoglobin levels in children aged 6 months and lowered the rate of anemia in comparison to the usual diets for this age. Regarding changes in growth, studies have been performed replacing the usual diets of animals with edible insects such as *HI* [35,36,38] and *GS* [49], measuring their fattening and weight increase, and two of them reported significant changes in weight gain [35,49]. Meanwhile, human studies [43,44,45] showed no significant differences in weight gain improvements between children treated with edible insects and children treated with cereals. This lack of improvement might be associated with the age of participants (<5 years) and various external factors, including difficulties in administering the food and poor adherence to treatment [45,63]. According to social development goals (SDGs), European countries are making efforts towards more sustainable alternative proteins and, in terms of accessibility, edible insects can offer new opportunities to underdeveloped countries [64].

Nutritional assessment of the fat contained in edible insects can play a positive role in feeding, given that they are rich in unsaturated fatty acids, especially polyunsaturated fatty acids (PUFAs) [65] and are especially low in cholesterol [66]. Edible insects contain n-3 PUFA. The increased demand for the omega-3 fatty acids eicosapentaenoic acid (EPA) and docosahexaenoic acid (DHA) has led science and industry to seek alternative methods to sustainably produce these essential fatty acids without relying on over-exploited wild fisheries [67], and edible insects may play a key role. Moreover, a diet that is mainly based on plant food products (common beans, wheat, soybeans, rice, and maize) could increase the risk of deficiencies in vitamin B12, EPA, and DHA [68]. Edible insects can be used to enrich diets, especially plant-based diets based on cereal proteins poor in essential AAs such as lysine, threonine, and tryptophan [69]. In addition, edible insects can be an alternative to proteins from traditional livestock (pork and beef), which have been directly related to increased cardiovascular and stroke risks. In animal studies, the positive effects of edible insect intake have been associated with a decrease in hepatic lipid droplets [33], a reduction [33] or non-increase [38,42] in plasma inflammatory biomarkers (ALT, AST, ALP) [33,38,42], decreases in plasma TNF-l levels [46], blood triglycerides [34,36], blood total cholesterol [36], and blood pressure [40], and an increase in blood serum hemoglobin levels [36,39]. *GbG* intake was found to have a positive effect on plasma glucose levels [41,42] and to reduce LDL-cholesterol and hepatocellular serum biomarkers [41,42]. Therefore, *GbB* can be used as a natural antioxidant, anti-lipidemic, functional food, and in the treatment of diabetes [41]. The therapeutic role of bioactive peptides (BAPs), may in part explain some physiological effects described in our review [70]. BAPs have been identified as edible insect protein hydrolysates/peptides, and their presence has been shown to be similar or higher than that of other dietary proteins in plants and animals. Further research on the BAPs derived from edible insects may reveal novel peptide sequences that may be more potent and/or bioavailable in comparison to BAPs from more conventional dietary proteins [70]. The interest of further pursuing research in the area of BAPs derived from edible insects may lead to the discovery of novel peptide sequences which may be more potent and/or more bioavailable than BAPs generated from more conventional dietary proteins. According to other reviews [71,72], research with insects tested in vivo and in cellular models displayed radical scavenging or metal ion chelation properties as well as the ability to modulate glutathione S-Transferase and catalase. It has been proposed that these activities, which are concentration-dependent, have beneficial antioxidant effects.

Insect protein has the potential to be an ecological, high-quality solution to meet future protein demands, and some insect proteins have proven equivalent or superior to soy protein in terms of nutritional value [47]. Gasco et al. [38] and Bovera et al. [36] described similar or better crude protein digestibility coefficients for diets with edible insect substitution (*HI* and *TM)* than for a soybean-based control diet. In human studies, edible insects showed high amounts of essential nutrients, and 77–98% of edible insect protein has been found to have high digestibility, depending on the species [73]. Protein is an essential macronutrient that is highly important for the structure of skeletal muscle, providing nitrogen and AAs [74]. In general, the availability of AAs stimulates muscle protein synthesis, which is necessary for the creation of skeletal muscle mass [75]. One study in humans [47] reported that supplementation with *AD* (insect inclusion rate of 6.25%) increased concentrations of blood EAA, BCAA, and leucine, similar to the effects of corn soybean meal. Animal protein supplementation was found to produce greater gains in muscle mass and strength compared to other plant sources such as soybean meal [48]. A protein intake of 0.8–1.6 g/kg/day is necessary to maintain protein balance and prevent muscle mass loss [76] in most individuals, but especially in older adults with a low daily energy intake. Older adults tend to consume less than the recommended amount of protein, often due to hyporexia, increasing their risk of fragility [77].

Insects also contain relevant levels of insoluble fiber derived from the exoskeleton, mainly in the form of chitin, which could have a positive impact on gastrointestinal health [78]. This insoluble fiber has been shown to exert antimicrobial, antioxidant, anti-inflammatory, anti-cancer, and immunostimulatory activities [79]. The chitin and chitosan content is between 4.3–7.1% and 2.4–5.8%, respectively, of the dry weight of whole crickets [80]. Supplementation with *TM* flour (inclusion level of 7.5%) had positive effects on intestinal microbiota growth (*Clostridium, Oscillospira, Ruminococcus, Coprococcus,* and *Sutterella*) and improved intestinal health in chickens [37]. In humans, *GS* flour intake resulted in a 5.7% increase in probiotic bacteria (*Bifidobacterium animalis)* in comparison to the same diet without this edible insect [46].

Studies have been published on allergic reactions to different insects and their components and on their cross-reactivity with crustaceans [81]. The main insect allergens were reported to be tropomyosin and arginine kinase, and this reactivity could not be eliminated by thermal treatment or digestion [81]. For instance, Barennes et al. [82] described allergic symptoms in 7.6% of frequent insect consumers, including individuals with allergies to dust mites and/or crustaceans, mainly attributed to the chitin from the exoskeleton [14].

Insects can appear as processed foods, serve as a supplement in animal feed or be used in human food to replace traditional ingredients, as in the case of margarine, milk, or burgers among others [83,84]. Pilot scale processing trials identified the potential of classical margarine technologies to transform insect lipids (*HI* and *TM*) into spreadable products with high fat content (more than 80%) and appropriate product coloring (yellowish) [83]. Substitution of 75% lipids in margarine resulted in a product with an environmental impact that was higher in comparison to conventional margarine, but lower in comparison to butter [85]. Other researchers developed an alternative to bovine milk from *TM* [84] that contained 5.76% fats and 1.19% proteins and represented 59.1% of the environmental burden of standardized bovine milk [84]. The addition of 10% cockroach flour in white bread formulation led to a protein increase of 133% (from 9.7% to 22.7%) and a fat reduction of 64.53%. Megido et al. [86] prepared different formulations of hamburger (beef hamburger, lentil hamburger, and lentil and beef hamburger with 50% insects (*TM*)). Other applications in the insect food industry are as emulsifiers. Higher emulsifying activity index (EAI) values *versus* other proteins and improved functional properties demonstrate the potential of cricket protein hydrolysates as a source of functional alternative proteins in food ingredient formulations [85]. The edible insect market is an emerging economic sector driven by strong market demand and supported by academic research and innovation in private sectors (from processing to selling).

Two aspects should be considered in the comparison of studies in the health dimension. First, consensus is required on the terminology used to facilitate the understanding of results when comparing different studies. Second, more long-term interventions are needed in humans to elucidate the effects on health, given that the average length of interventions is 2 months in animals and 5 months in humans (>2 months in only 2 of the 6 human studies). Regarding the terminology, the four indicators used in this systematic review can be recommended for human studies on the effect of insect intake: (a) the daily food portion used in interventions with insects, (b) insect inclusion level CFP, (c) protein inclusion level of CFP, and (d) protein of CFP per day. The insect inclusion level (g insect/100 g of product) is provided in four of the human studies [43,46,47,48], with inclusion levels of 1.8% and 9.37–14.9%, respectively, in two of these [43,46]. Vangsoe et al. [47,48] used lower insect inclusion levels of 0.04–7.6% in the form of isolated protein, which has a higher protein content than edible insect powder [81]. Hence, it is crucial to specify whether flour or isolate is used to avoid errors in comparisons. The way in which edible insects are administered is relevant because it may have an impact on the protein digestibility. The term “insect inclusion level” is widely used in animal studies, and 11 of the 12 studies described values ranging between 0.01% and 28.3% for whole edible insect powder and between 0.0005% and 0.05% for the edible insect components glycosaminoglycan [41,42] and chitin/chitosan [49]. The protein quantity of the CFP per day describes the amount of protein provided in the intervention, and this information would be even more valuable if it included the amount of protein provided in the CFP from insects, given that the amount of insect in many CFP compositions is very low compared to other ingredients [43,44]. The protein inclusion level has the potential to allow calculation of the percentage of insect protein with respect to the amount of protein in the CFP.

With regards to the environmental dimension, the increasing need for food production is hampered by the shortage of land for agricultural production for land [87]. Humans currently consume around 40% of the biomass on land and coastlines, and the massive demand for animal proteins, recognized as one of the leading causes of climate change, has created the need for protein alternatives to protect the welfare of future generations [88]. The high nutritional value of edible insects and their protein content make them excellent alternatives to conventional protein sources for animal feed [89]. The environmental impact of current protein sources is very high, contaminating surface waters, spreading pathogenic microorganisms and chemical pollutants, emitting GHG, and causing deforestation [90]. In contrast, insect farming reduces the environmental footprint [91] and the use of pesticides and water [92], and it offers more efficient food conversion. For example, cattle and pigs are considered responsible for 18% of all GHGs, with a major impact on global warming. In terms of sustainability, the waste generated in insect farms is minimal in comparison to that produced by stockbreeding, which is responsible for around 10% of GHG emissions in Europe [93]. The WFP is 79% lower for the production of edible insects than for raising cattle [55], and its LU requirements are 61% lower in comparison to traditional livestock production [93]. The food conversion ratio, i.e., the kg of food needed to make 1 kg of edible weight, is also much lower for edible insects than for pigs, chickens, and cattle [11,94]. Therefore, insects could be a more environmentally and economically sustainable source of protein. As an example, the harvested and processed black soldier fly larvae, valued at around US$200 per ton, can also be more economically transported than manure (valued at USD 10–20 per ton) [95]. In the present systematic review, we highlight the importance of edible insects as food, feed, or waste utilization farming, comparing four environmental impacts (LU, GHG, EU, and WFP) with the FU per kg edible protein and comparing these indicators to those for more traditional proteins, such as those from livestock. Van Raamsdonk et al. [96] discussed the issues related to insects as feed material and showed that insect farming offered a smaller environmental footprint, reduced pesticide use, more efficient food conversion, and a lesser water requirement in comparison to traditional livestock. Furthermore, the conversion efficiency of ingested food was estimated to be 58–85% lower in edible insects *versus* pork and beef and 17% lower *versus* poultry [96] (Table 5).

A better environmental performance is obtained with waste-fed insects [50,52] than with non-waste-fed insects [53,54]. For human food consumption, the edible insect farm uses a mixed diet (oats, carrots, wheat) that almost doubles LU and GHG values [97]. However, the waste generated in an insect farm is minimal in comparison to that produced by stockbreeding, which is responsible for around 10% of GHG emissions in Europe [94].

**Figure 2:**
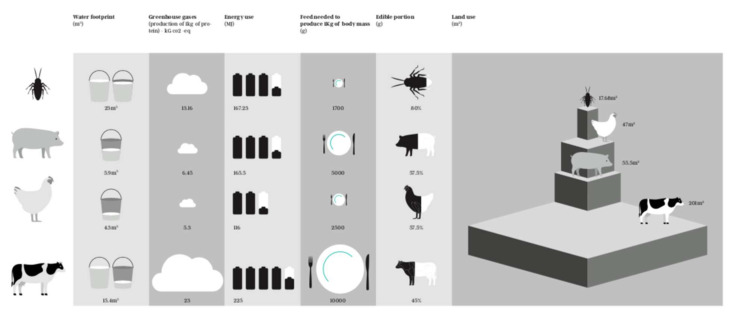
Resource use and environmental impact parameters of insect farming *versus* the production of other livestock (Data on resource use and environmental obtained from Table 3 and Table 4).

The production of edible insects for human consumption is responsible for around 95% less GHG emissions and LU and 62% less EU in comparison to beef production [93]. The environmental benefits of insects are much higher compared with other livestock (pork and poultry), achieving reductions of 90% in GHG, 61% in LU, and 56% in EU [93] (Figure 2).

**Figure 3:**
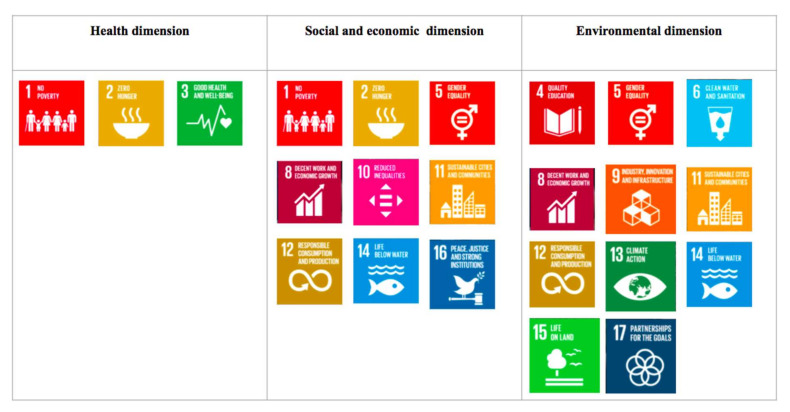
Edible insects and Sustainable Development Goals according to the three dimensions of food sustainability.

Further research is needed on EU. For example, EU and related GHG emission may be lesser if insects are used for composting [98]. EU per kg of protein is currently higher for insect meal than for soybean or fish meal [53]. It is recommended to lower EU by using more efficient heating and air-conditioning devices and by adequately insulating production facilities, besides designing automated measures for the separation of pupae and residue substrates [52]. Reducing WFP requires challenging research to conceive and design alternative cleaning measures and/or rearing vessels with a more favorable volume/surface area ratio [52]. Implementing innovative and sustainable food production strategies such as insect farming may contribute to several SDGs, which are themselves interconnected [64], given the importance in food sustainability as defined by Béné et al. [99] (Figure 3).

Other considerations to be taken as part of the drive towards food sustainability are the acceptability, accessibility, and affordability of edible insect products, which are key to their introduction into the dietary habits of Western societies [99]. In developed countries, entomophagy has been considered a primitive behavior and relegated to rural environments [82], and industrialized populations associate insects with fear and disgust [100]. The exclusion of entomophagy is above all a cultural issue, and it is hard to persuade individuals to accept the practice [101]. The acceptability by populations of food products with edible insects may be difficult in countries where they are not traditionally consumed [102]. It is therefore necessary to search for attributes that could support their popularization [103,104].

## 5. Conclusions

This systematic review contributes specific information on the potential health benefits offered by edible insects. In animal and human studies, doses up to 7% of edible insect inclusion level significantly improved the live weight, reduced levels of triglycerides, cholesterol, and blood glucose, and increased microbiota diversity (*versus* control diet) in animals, and significantly improved gut health, reduced systemic inflammation (*versus* control diets), increased blood concentrations of essential and branched-chain amino acids, and slowed digestion (*versus* whey treatment) in humans. Comparisons among studies could be facilitated if researchers consistently use three nutritional composition indicators (insect inclusion level, insect protein inclusion level, and total protein inclusion level). Environmental indicators (land use, water footprint, and greenhouse gas emissions) were 40–60% lower for the feed and food of edible insects than for traditional animal livestock, thereby diminishing the carbon footprint. Edible insects contribute to the circular economy because they can use the food and feed waste generated by animals and humans, adding value to these waste products. However, edible insect farming should be more efficient in terms of EU and the need to introduce renewable energy. An important future goal would be to increase the percentage of insect inclusion level in human and animal studies, to make a significant contribution to its application as novel supplementation for health. Moreover, the doses should be comparable to those more widely used for vegetable or animal protein sources. It is essential to increase the social acceptability of edible insects, the main barrier to their introduction into Western diets. Long-term studies are required to elucidate the effect of edible insects on human health, and more data are needed on environmental indicators of the use of edible insects for human food consumption. Food systems should explore alternative sources of proteins, and edible insects are an opportunity for the food industry to improve environmental indicators, with the associated economic benefits, social impact, and global enhancement of planetary health.

## Figures and Tables

**Table 1:** Risk of bias in animal and human studies on the health effects of edible insects.

Animal Studies	Selection Bias	Performance Bias	Detection Bias	Attrition Bias	Reporting Bias	Others
Kim et al. [32]	-	-	-	-	-	-
Seo et al. [33]	-	-	-	-	?	?
Bergmans et al. [34]	?	-	-	-	-	-
Dabbou et al. [35]	-	-	-	-	-	?
Bovera et al. [36]	-	-	-	-	-	?
Biasato et al. [37]	-	-	-	-	-	-
Gasco et al. [38]	-	-	-	-	-	-
Agbemafle et al. [39]	-	-	-	-	-	-
Pessina et al. [40]	-	-	-	-	-	?
Ahn et al. [41]	-	-	-	-	-	?
Ahn et al. [42]	-	-	-	-	-	?
**Human Studies**	**Selection Bias**	**Performance Bias**	**Detection Bias**	**Attrition Bias**	**Reporting Bias**	**Others**
Skau et al. [43]	-	-	-	?	-	-
Bauserman et al. [44]	-	-	-	?	-	-
Nirmala et al. [45]	-	?	+	?	-	?
Stull et al. [46]	-	-	-	-	-	-
Vangsoe et al. [47]	-	-	-	-	-	-
Vangsoe et al. [48]	-	?	-	-	-	-

Summary of risk of bias: review of the opinions of the different authors on each element of bias risk for each study. The minus sign (-) indicates low risk of bias, plus sign (+) high risk of bias, and question mark (?) unclear risk.

**Table 2:** Effect of edible insects on animal health.

Author, Year, Country	Type of Animal, Sample Size (Male/Female), Age	Duration (Days)	Insect	Intervention (n)	Insect Inclusion Level of CFP (g/100 g Expressed in %)	Variables/Outcomes	Results
Kim et al., 2016 (Korea) [32]	C57BL/6J mice 40 (40/-); 7 weeks	56	*Allomyrina dichotoma* larvae	All groups (a–e) start with: 8 weeks (diet-induced obesity): HFD, 60% fat (obese mice). (a) HFD 60%+ 1 μL of 20% DMSO ^1^ (n = 10) (b) HFD 60% + 1 μL of ALLD ^1^ (10 mg/mL) (n = 10) (c) LFD 10% (d) LFD, 10% + 1 μL of 20% DMSO ^1^ (n = 10) (e) LFD, 10% + 1 μL of ALLD ^1^ (10 mg/mL) (n = 10)	(b) 1% ^2^	***Appetite control*** (food intake and body weight) ***Metabolic traits*** (inflammatory indicators)	Food intake and body weight were reduced (b,e) compared to (a,b) respectively (S). ADE resulted in strong reduction of ER stress compared to (a) (S).
Ahn et al., 2016 (Korea) [42]	Wistar rats, 50 (50/-), 14 weeks	30	*Gryl-* *lus bimaculatus*	(a) Control + HFD (n = 10) (b) *GbG*5 + HFD (n = 10) (c) *GbG*10 + HFD (n = 10) (d) Pravastatin + HFD (n = 10) (e) Chitosan + HFD (n = 10)	(b) 0.0005 *GbG* (c) 0.001 *GbG*	***Metabolic traits*** (blood parameters and blood pressure)	Weight of abdominal and epididymal fat, AST, ALT, total cholesterol, and glucose were lower after (b,c) compared to (a) (S). Blood pressure was similar after b,c compared to (a) (NS). Anticoagulant and antithrombotic effects were seen: platelet, thrombin time, prothrombin time and factor I were increased with (b,c) treatment (S). CRP levels of (b,c) decreased compared to (a) (S).
Seo et al., 2017 (Korea) [33]	BALB/c mice, 35 (35/-), 5 weeks	42	*Tenebrio molitor* larvae	(a) ND (10% fat) (n = 7) (b) HFD (60% fat) (n = 7) ^3^ (c) HFD (60% fat) with TML (n = 7) ^3^ (d) HFD (60% fat) with TML (n = 7) ^3^ (e) HFD (60% fat) with 3000 mg/kg of yerba mate (n = 7) ^3^	(c) 0.01% (d) 0.30%	***Metabolic traits*** (weight gain, fat mass, hepatic steatosis, blood parameters)	Body weight gain, epididymal white adipose tissue size and volume decreased after (c,d) compared to (b) (S). Mean adipocyte volume was reduced after (d) compared to (b) (S) Hepatic lipid droplets, plasma ALT and AST levels, visceral fat were reduced after (c,d) compared to (b) (NS).
Dabbou et al., 2018 (Italy) [35]	Ross 308 CD1-IGS broiler chicken, 256 (256/-) ND	35	*Hermetia Illucens* larvae	(a) HI0 (n = 64) (b) HI5 (n = 64) (c) HI10 (n = 64) (d) HI15 (n = 64)	(a) 0% (b) 5% (c) 10% (d) 15%	***Growth performance*** (weight gain, feed intake) ***Metabolic traits*** (blood parameters, inflammatory indicators) ***Intestinal morphology***	Dietary HI inclusion (b–d) positively influenced growth performance up to 10%, in terms of improved live weight and daily feed intake during the starter period (S). At 10, 24, and 35 days of age, live weight showed a linear and quadratic response to HI meal with a maximum observed for (c) (S). HI showed a linear response (*p* = 0.002) to increases up to d) for blood or serum glutathione peroxidase (NS). Intestinal villus height was lower, crypt depth was greater, and villus height-to-crypt depth ratio was lower after (d) compared to (a–c) (S).
Bovera et al., 2018 (Italy) [36]	Hy-line Brown hens, 162 (-/162), 16 weeks	140	*Hermetia Illucens* larvae	(a) Control group: corn-soybean meal-based diet (n = 54) (b) HI25(n = 54) (c) HI50 (n = 54)	(b) 7.3% (c) 14.6%	***Metabolic traits*** (blood parameters) ***Crude protein digestibility***	Serum cholesterol and triglyceride levels were reduced after (b,c) compared to (a) (S). Serum globulin levels were higher after (c) compared to (a,b) (S). Crude protein digestibility was the highest (*p* < 0.05) in (a), followed by (b,c) (NS).
Biasato et al., 2018 (Italy) [37]	Label Hubbard hybrid Chickens, 140 (-/140), 43 days	140	*Tenebrio molitor larvae*	(a) Control group: corn-soybean-gluten meal-based diet (n = 70) (b) TM 7.5 (n = 70)	(b) 7.5%	***Intestinal morphology***	Small intestine revealed similar villus height, crypt depth, and villus height crypt depth ratio between (a,b) (NS).
Gasco et al., 2019 (Italy) [38]	Crossbred rabbits, 200 (ND), 36 days	41	*Hermetia Illucens/Tenebrio molitor* larvae	(a) Control group: 1.5% soy-bean oil (n = 40) (b) H50 (n = 40) (c) H100 (n = 40) (d) T50 (n = 40) (e) T100 (n = 40)	(b) 0.75% (c) 1.5% (d) 0.75% (e) 1.50%	***Growth performance*** (feed intake and body weight) ***Metabolic traits*** (blood parameters) ***Crude protein digestibility*** ***Intestinal morphology***	Weight gain and feed intake was affected similarly after (a–e) (NS). Including insect lipids in rabbit diets did not influence AST, ALT, or ALP enzyme activities. Blood variables were affected similarly after (a–e) (NS). Crude protein digestibility was affected similarly after (a–e) (NS). Villi height, crypt depth, and their ratio were affected similarly after (b–e) compared to (a) (NS).
Agbemafle et al., 2019 (Ghana) [39]	Sprague–Dawley rats, 66 (66/-), 21 days	35	*Acheta domesticus/Rhynchophorus phoenicis fabricius*	(a) Normal rats + Casein + ferrous sulfate (n = 8) (b) MD ^4^ (5% protein) + low protein -Fe (n = 8)-negative control (c) MD ^4^ (5% protein) + *S.torvum* (26.7) (n = 8) (d) MD ^4^ (5% protein) + AD + *S. torvum* (n = 8) (e) MD ^4^ (5% protein) + Protein Fe sufficient (n = 8)-positive control (f) MD ^4^ (5% protein) + AD (n = 8) (g) MD^4^ (5% protein) + RF (n = 8)	(d) 15.4% (f) 28.3%	***Growth performance*** (body weight recovery, fat mass) ***Metabolic traits*** (blood parameters)	After malnourished treatment, weight gain, bone mineral content and lean and fat mass increased similarly after (d,f,g) compared to (e) (NS). Hb increased after (f,g) compared to (a) (NS).
Lokman et al., 2019 (Malaysia) [49]	Cobb500 broiler chickens, 100 (150/-), 150 days	42	*Gryllodes sigillatus*	(a) Control: Baseline diet (n = 30) (b) Baseline diet + 0.5 g/kg cricket chitin (n = 30) (c) Baseline diet + 0.5 g/kg cricket chitosan (n = 30) (d) Baseline diet + 0.5 g/kg shrimp chitin (n = 30) (e) Baseline diet + 0.5 g/kg shrimp chitosan (n = 30)	(b) 0.05% chitin (c) 0.05% chitosan	***Growth performance*** (body weight, feed intake and fat mass)	Body weight and feed intake improved after (b) compared to (c) (S). Body weight of a) accumulated more fat compared (b–e) (S).
Bergmans et al., 2020 (USA) [34]	Mice, 65 (65/-), 3 weeks	66	*Gryllodes sigillatus*	(a) Control group: Standard adult diet 2018 (n = 10–12) ^5^ (b) HD+Cricket-based diet (n = 10–12) ^5^ (c) HD +Milk-based diet, (n = 10–12) ^5^ (d) HD+Peanut-based diet (n = 10–12) ^5^		***Growth performance*** (body weight recovery) ***Metabolic traits*** (blood parameters)	After malnourished treatment and recovery diets, there was an increment weight (34%) after (b) compared to (a) (NS). Triglycerides were reduced (47%) after (b) compared to (a) (S). After six weeks on recovery protein diets, there were no differences in the splenetic expression of select inflammatory genes among (a–c) (NS).
Pessina et al., 2020 (Brazil) [40]	Spontaneously hypertensive rats (SHR) 24 (24/-) and age-matched WKY rats (controls) 18 (18/-), 9 weeks	28	*Tenebrio molitor* larvae	(a) SHR SD (n = 8) (b) SHR SD + TM (n = 8) (c) SHR SD + captopril (n = 8) (d) WKY SD (n = 6) (e) WKY SD + TM (n = 6) (f) WKY SD + captopril (n = 6)	(b) 0.29% (e) 0.29%	***Metabolic traits*** (blood parameters, blood pressure and inflammatory indicators)	Systolic BP, heart rate, and coronary perfusion pressure were reduced after (b,c) compared to (a) (S). Rat brain slices of SHR were more resistant to oxidative stress and contained lower levels of inflammatory cytokines, with no effect on vascular and liver enzyme activities (S).
Ahn et al., 2020 (Korea) [41]	BKS.Cg-m+/+Leprdb, heterozygous (DB-Hetero, normal) (db/+) male mice (11/-), 12 weeks and homozygous (DB-Homo, diabetes) (db/db) male db mice, 33 (33/-), 12 weeks	30	*Gryllus bimaculatus-* *tus*	(a) Normal Hetero (DB-Hetero) (n = 11) (b) Control Homo (DB-Homo) (n = 11)-negative control (c) DBHomo + 5 mg/kg treatment of CaG (CaG5) (n = 11) (d) DB Homo + 5 mg/kg treatment of *GbG* (*GbG*5) (n = 11) (e) DBHomo + 10 mg/kg treatment of metformin (n = 11)-positive control	(d) 0.0005% *GbG*	***Metabolic traits*** (blood parameters, and antioxidant activity)	Capacity to reduce glucose, ALT, AST, ALP, LDL-cholesterol and BUN levels increased after (d) compared to (b) (S). Antioxidant activities (catalase, SOD and GPX) increased after (d) compared to (b) (S).

^1^ Injection at week 17 and week 18; ^2^ g/100 mL; ^3^ induced obesity; ^4^ malnourished diet; ^5^ Start with initial weaning diet 2020 for 10 days; S: significant; NS: non-significant; CFP: complementary food product; HFD: high fat diet; HD: Hypoprotein diet; DMSO: dimethyl sulfoxide; ALLD: *Allomyrina dichotoma* larvae; AD: *Acheta domesticus*; LFD: low-fat diet; ER: endoplasmic reticulum; ND: normal diet; TM:*Tenebrio molitor*; ALT: alanine transaminase; AST: aspartame transaminase; GS: *Gryllodes sigillatus*; HI: *Hermetia illucens*; AD: Acheta domesticus; RF: Rhynchophorus phoenicis fabricius; MD: malnourished; Fe: Iron; SD: standard diet; BP: blood pressure; DB: diabetes; CaG: *Dung beetle* (*C. molossus*) glycosaminoglycan; GbG: *Grillodes bimaculatus* glycosaminoglycan; LDL: low density lipoprotein; ALP: alanine transaminase; SHR: spontaneously hypertension rats; WKY: Wistar Kyoto Rats; CRP:C-reactive protein; BUN: Blood urea nitrogen; SOD: Superoxide dismutase; GPX: Glutathione peroxidase.

**Table 3:** Effect of edible insects on human health.

Author, Year, Country	Type of Study	Subjects, Sample Size (Male/Female), Age	Duration (Days)	Insect	Intervention	Daily Food Portion of Intervention with Insects	Insect Inclusion Level of CFP (g/100 g Expressed in %)	Insect Inclusion of CFP (Expressed in g) for Each Age Group	Protein Inclusion Level of CFP: g/100 g (Expressed in %)	Protein of CFP Per Day (Expressed in g).	Variables/Outcomes	Results
Skau et al., 2015 (Cambodia) [43]	Randomized, single-blinded trial	Infants, 419 (220/119), 6 months	270	*Haplopelma species*	(a) WF: Rice-based ^1^ CFP with small fish and edible spiders (n = 106) (b) WF-L ^1^: Rice-based CFP with small fish (n = 104) (c) CSB++ ^1^: Fortified corn-soy blend product (n = 103) (d) CSB+ ^1^: Fortified whole-soy (n = 106)	1. Infants 6–8 months: 50 g. 2. Infants 9–11 months: 75 g. 3. Infants 12–15 months: 125 g.	a1,a2,a3 = 1.8%	(a.1) 0.9 g (a.2) 1.35 g (a.3) 2.25 g	(a)15.4% (b)12.6% (c)16.8% (d)14.6%		***Growth performance*** (food intake, body weight) ***Metabolic traits*** (blood parameters)	Total weight increases in (a), (b) compared to vs c) (NS). Similar growth observing no differences between (a–d) groups (NS) FFM no differences were observed between (a,b) (NS). Plasma ferritin, sTfR, and hemoglobin concentration no differences were observed between (a–d) (NS). Total weight increase in (a,b) compared to (c) (NS).
Bauserman et al., 2015 (Democratic Republic of Congo) [44]	Cluster- randomized controlled trial	Infants, 222 (113/109), 6 months	540	*Caterpillar*	(a) Usual diet ^2^ (n = 110) (b) Caterpillar ^2^ cereal. (n = 110)	Infants 6–12 months of age: 30 g Infants 12–18 months: 45 g				(b.1) 6.9 (b.2) 10.3	***Growth performance*** (body weight recovery) ***Metabolic traits*** (blood parameters)	Stunting prevalence, no differences were observed between (a,b) (NS). Fe: no differences were observed between (a,b) (NS). Hb increased in (b) compared to (a) anemia decreased in (b) compared to (a) (S).
Nirmala et al., 2017 (Indonesia) [45]	Non-randomized controlled trial	Infants, 23 (12/11), 1–5 years	45	*Rhynchophorus ferrugineus*	(a) Usual diet (n = 10) (b) Sago worm inclusive diet (n = 13)	2 pieces of 50 g			(b) 9.70%	(a) 3.9 ±1.7 (b) 5.9 ± 1.7	***Growth performance*** (body weight)	Weight and height no changes were observed between a) and b) (NS)
Stull et al., 2018 (USA) [46]	Double-blinded randomized crossover trial	Healthy adults, 20 (9/11), 18–65 years	14	*Gryllodes sigillatus*	(a) Control breakfast meal (n = 10) (b) Cricket breakfast meal (n = 10)	Shake + pumpkin muffin (160 g)	(b) 14.9% Shake; 9.37% Muffin		(b) 14.78%	(a) 9 (b) 21.67	***Gut microbiome composition*** ***Metabolic traits*** (inflammatory indicators)	Bifidobacterium animalis increased 5.7 more in (b) compared to (a) (S) Plasma TNF-α decreased b) compared to (a) (S).
Vangsoe et al. A 2018 (Denmark) [47]	Randomized, controlled, single-blinded trial	Healthy young adults, 18(18/-), 18–30 years	56	*Alphitobius diaperinus*	(a) Isocaloric carbohydrate bar (n = 9) (b) Insect protein bar (n = 9)	2 bars a day	(b) 0.04% ^3^			(a) 7.2 (b) 8	**Changes in muscle mass composition and** **strength**	Morphological adaptations such as hypertrophy or muscle strength show no changes in (a) compared to (b) (NS).
Vangsoe et al. B 2018 (Denmark) [48]	Randomized, cross-over study	Healthy young adults,6 (6/-), 18–30 years	1	*Alphitobius diaperinus*	(a) Drink placebo (water) (b) Drink whey isolate (c) Drink soy isolate (d) Drink insect isolate	400 mL per day	(d) 7.6% ^4^	(d) 30.5 g isolate powder		(b) 25 g (c) 25 g (d) 25 g	***Crude protein digestibility***	Blood concentrations of EAA, BCAA and leucine increased in (b–d) compared to (a) over a 120 min period (S). Slowly digested (d) compared to (b,c) (S).

^1^ Severe malnutrition; ^2^ stunning rates; ^3^ isolated powder 82% protein; ^4^ 30.5 g/400 mL; S: significant NS: non-significant; CFP: complementary food product; WF: win food; WF-L: win food lite; CSB: corn soy blends; FFM: fat free mass; sTfr: soluble transferrin receptor; Fe: iron; Hb: hemoglobin; TNF-α: plasma tumor necrosis factor-alpha; AA: amino acid; EAA: essential amino acids; BCAA: branched-chain amino acids.

**Table 4:** Effect on environmental indicators of edible insects consumed as animal feed and human food.

	Animal Feed Consumption (kg Edible Protein)								Human Food Consumption (kg De Protein)				
	Waste-Feed Insects				Non Waste Insects								
Insect	Author, Year, Country	Land Use (m^2^)	GHG (Kg CO_2_ eq)	Energy Use (MJ)	Author, Year, Country	Land Use (m^2^)	GHG (Kg CO_2_ eq)	Energy Use (MJ)	Author, Year, Country	Water Footprint (m^3^)	Land Use (m^2^)	GHG (Kg CO_2_ eq)	Energy Use (MJ)
***Tenebrio molitor larvae***					Thévenot et al., 2018 (France) ^F,J^ [53]	6.35	5.77	217.37	Oonincx et al., 2012 (USA) ^F^ [7]		17.68	13.16	167.23
									Miglietta et al., 2015 (Italy) ^F,I^ [55]	23			
***Musca domestica larvae***	Van Zanten et al., 2015 (Netherlands) ^A,J^ [50]	0.07	1.43	18.98									
***Hermetia illucens larvae***	Salomone et al., 2016 (Italy) ^B,I^ [51]	0.05	2.1	15.1									
	Muys et al. 2014 (UK) ^C,I^ [52]	0.06 ^D^ 0.19 ^E^	2.1	15.1									
***Acheta domesticus***					Halloran et al., 2017 (Denmark) ^F,I^ [54]	3.97 ^G^ 2.63 ^H^							

A: Poultry manure; B: food waste; C: brewery waste; D: manual harvest; E: automatic harvest; F: mixed diet; G: current situation; H: future scenario; system boundaries: (I: from cradle to farm and J: from cradle to meal).

**Table 5:** Environmental indicators of traditional livestock animals for food.

Traditional Livestock Animals for Food (kg de Protein)					
Animal	Author, Year, Country	Water Food Print (m^3^)	Land Use (m^2^)	GHG (Kg CO_2_ eq)	Energy Use (MJ)
**Pork**	Vries and de Boer. 2010 (Netherlands) ^F^ [97]		47–64	21–53	95–236
	Miglietta et al. 2015 (Italy) ^F^ [55]	57			
**Chicken**	Vries and de Boer. 2010 (Netherlands) ^F^ [97]		42–52	18–36	80–152
	Miglietta et al. 2015 (Italy) ^F^ [55]	34			
**Beef**	Vries and de Boer. 2010 (Netherlands) ^F^ [97]		144–258	75–170	177–273
	Miglietta et al. 2015 (Italy) ^F^ [55]	112			

F: Mixed diet.
